# Chemical profile, virtual screening, and virulence-inhibiting properties of *Sphagneticola trilobata* L. essential oils against *Pseudomonas aeruginosa*

**DOI:** 10.1038/s41598-025-94486-0

**Published:** 2025-04-08

**Authors:** Wafaa H. B. Hassan, Afaf E. Abdel Ghani, Esraa A. Taema, Galal Yahya, Mohamed E. El-Sadek, Basem Mansour, Mahmoud Saad Abdel-Halim, Asmaa M. Arafa

**Affiliations:** 1 Department of Pharmacognosy, Faculty of Pharmacy, Zagazig University, Zagazig, 44519 Egypt; 2 Department of Microbiology and Immunology, Faculty of Pharmacy, Zagazig University, Zagazig, 44519 Egypt; 3 Department of Medicinal Chemistry, Faculty of Pharmacy, Zagazig University, Zagazig, 44519 Egypt; 4 Department of Pharmaceutical Chemistry, Faculty of Pharmacy, Delta University for Science and Technology, International Coastal Road, Gamasa City, 35712 Egypt

**Keywords:** *Sphagneticola trilobata* L., GC/MS, *Pseudomonas aeruginosa*, Biofilm, Quorum sensing, Virtual screening, Drug discovery, Microbiology, Plant sciences

## Abstract

The escalating threat of microbial resistance underscores the urgent need for innovative solutions, including natural agents capable of attenuating virulence. This study explores the antimicrobial and anti-virulence potential of essential oils (EOs) derived from *Sphagneticola trilobata* against *Pseudomonas aeruginosa*. Through GC/MS analysis, volatile metabolites from the flower heads and leaves/stems of Egyptian *S. trilobata* were identified, revealing 43 and 62 components, respectively. Key compounds included α-phellandrene, α-pinene, D-limonene, and α-thujene. The Minimum Inhibitory Concentrations (MIC) of flower head and leaf/stem EOs against *P. aeruginosa* were 1.17% and 1.75% *v/v*, respectively. At sub-MIC doses (1/8th of the MIC), the EOs exhibited significant anti-virulence properties, including complete inhibition of protease activity and disruption of biofilm formation, which are crucial for bacterial survival and pathogenicity. Additionally, they effectively suppressed the expression of quorum sensing genes, which are essential for bacterial communication and virulence. Virtual screening of four major EO components (+)-(R)-limonene, (±)-α-pinene, α-phellandrene, and α-thujene against five critical protein targets involved in biofilm formation, quorum sensing, virulence, and protease activity in *P. aeruginosa* further supported their anti-virulence and antibiofilm actions, showing high affinity for these targets. These findings suggest that the EOs of *S. trilobata* hold great potential as natural virulence attenuating agents, particularly against biofilm-forming pathogens like *P. aeruginosa*.

**Supplementary Information:**

The online version contains supplementary material available at 10.1038/s41598-025-94486-0.

## Introduction

Medicinal plants represent the earliest known natural pharmacy, with herbal medicines and their essential oils (EOs) historically serving as key tools to combat microbial infections long before the discovery of antibiotics^1–4^. However, in recent years, microbial resistance has emerged as a silent pandemic, exacerbated by the slow development of new antibiotics and the increasing prevalence of pan-resistant pathogens^5–9^. This urgent global challenge has driven scientists to explore novel alternatives, including resistance-modifying agents. In this context, there is a renewed focus on natural sources, with researchers actively screening herbal medicines, EOs, and phytochemicals for their potential as effective resistance-modifying agents^10,11^. EOs are complex blends of volatile bioactive components, mainly terpenes and terpenoids. Until now, around 300 of the 3000 recognized EOs have been utilized in the food and pharmaceutical fields^12,13^.

*Sphagneticola trilobata* L. Pruski (*Wedelia trilobata*), a perennial creeping herb belonging to family *Asteraceae*, is widely cultivated as an ornamental plant. *S. trilobata* has various pharmacological properties as antimicrobial, insecticidal, antioxidant, antidiabetic, anticancer, and anti-inflammatory due to its content of lactones, sesquiterpenes, triterpenes and diterpenes^14,15^.

The EO of *S. trilobata* is primarily composed of *β*-Phellandrene, limonene, γ-terpinene, *β*-caryophyl lene and *α*-pinene^16^. Most of these components have implementations in the industries of pharmaceuticals, cosmetics, and food^17^. However, despite their potential, biological studies on *S. trilobata*’s EO remain limited, with most research focusing on its antimicrobial and antioxidant activities^14,16^. The antibacterial properties of *S. trilobata* EO have been demonstrated against a range of pathogens, including *Streptococcus mutans*, *Staphylococcus aureus*, *Bacillus subtilis*, *Microbacterium phlei*, *Escherichia coli*, *Sarcina lutea* and *P. aeruginosa*^16,18^. Notably, there is a lack of documented studies on the EOs derived from different organs of Egyptian *S. trilobata*, with only one report focusing on the flower heads’ EO^16^. To address this gap, the current study aimed to characterize and compare the volatile metabolites from different organs of Egyptian *S. trilobata* using GC/MS analysis. Additionally, the antimicrobial properties of the EOs were assessed against *P. aeruginosa*, one of the most troubling pathogens associated with arsenals of resistance mechanisms, prevalent in hospitals and notably pervasive in intensive care units (ICUs)^19^. It is implicated in a range of life-threatening infections in the ICU setting, including endocarditis, septicemia, urinary tract infections, cystitis, pneumonia, and surgical wound infections^20^. We examined the anti-virulence activity of Egyptian *S. trilobata* EOs for the first time. This encompassed assessing their impact on biofilm formation, protease secretion, and cell-to-cell communication *via* quorum sensing.

## Results

### Composition of *Sphagneticola trilobata* essential oils

**Fig. 1:**
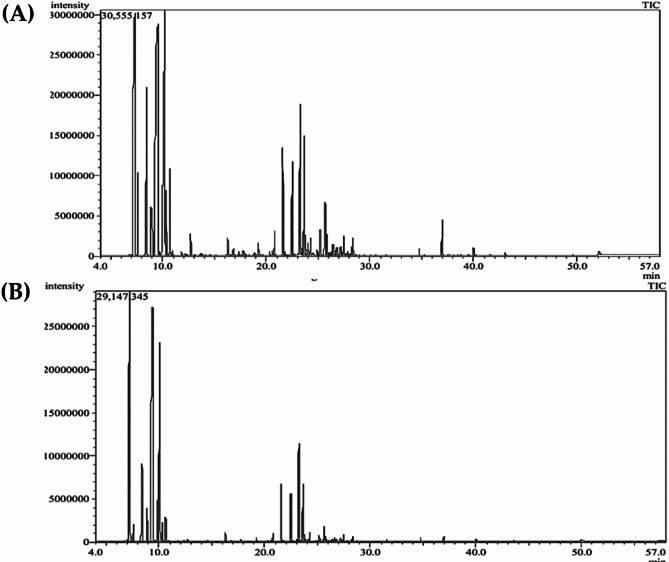
GC-MS chromatograms of *S. trilobata* EOs from collected leaves and stems mixture (**A**) and flower heads (**B**).

**Fig. 2:**
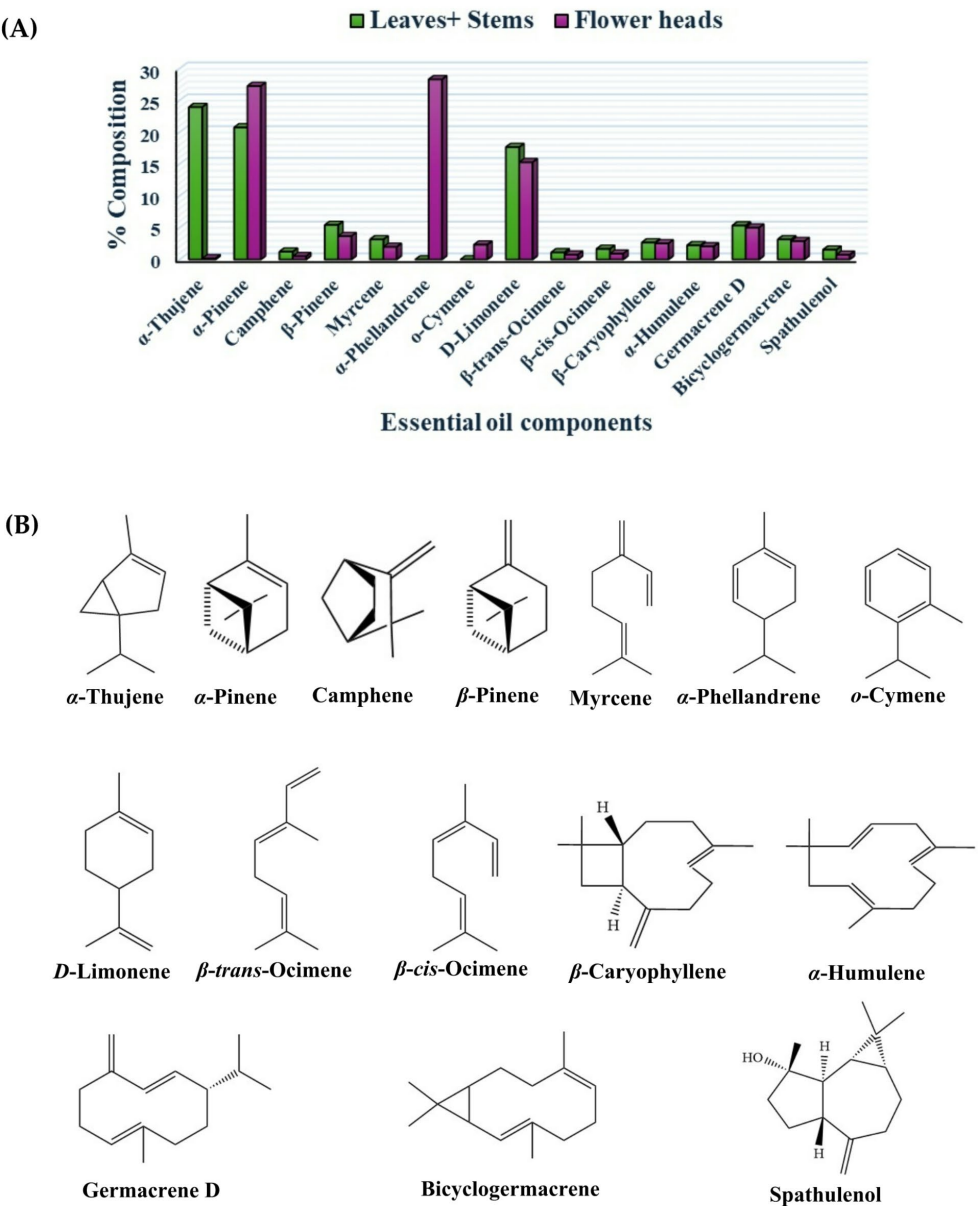
The main EOs components of *S. trilobata*. (**A**) Composition (%); (**B**) Chemical structure.

GC-MS profiling of *Sphagneticola trilobata* EOs from different organs led to the identification of 68 components: 62 components from the collected leaves and stems mixture and 43 of them from flower heads. The identified components are classified into eight chemical classes, representing 99.40% and 99.64% of the EO compositions in the collected leaves and stems mixture and flower heads, respectively (Fig. 1 and Supplementary Table S1). The main EOs components are displayed in Fig. 2. α-Thujene (23.94%), α-pinene (20.75%) and D-limonene (17.66%) are the main components of the EO from the collected leaves and stems mixture. On the other hand, the main EO components of flower heads are α-phellandrene, α-pinene, and D-limonene with percentages of 28.30%, 27.25% and 15.28%, respectively.

### Microbiological evaluation and effect on bacterial quorum sensing

**Fig. 3:**
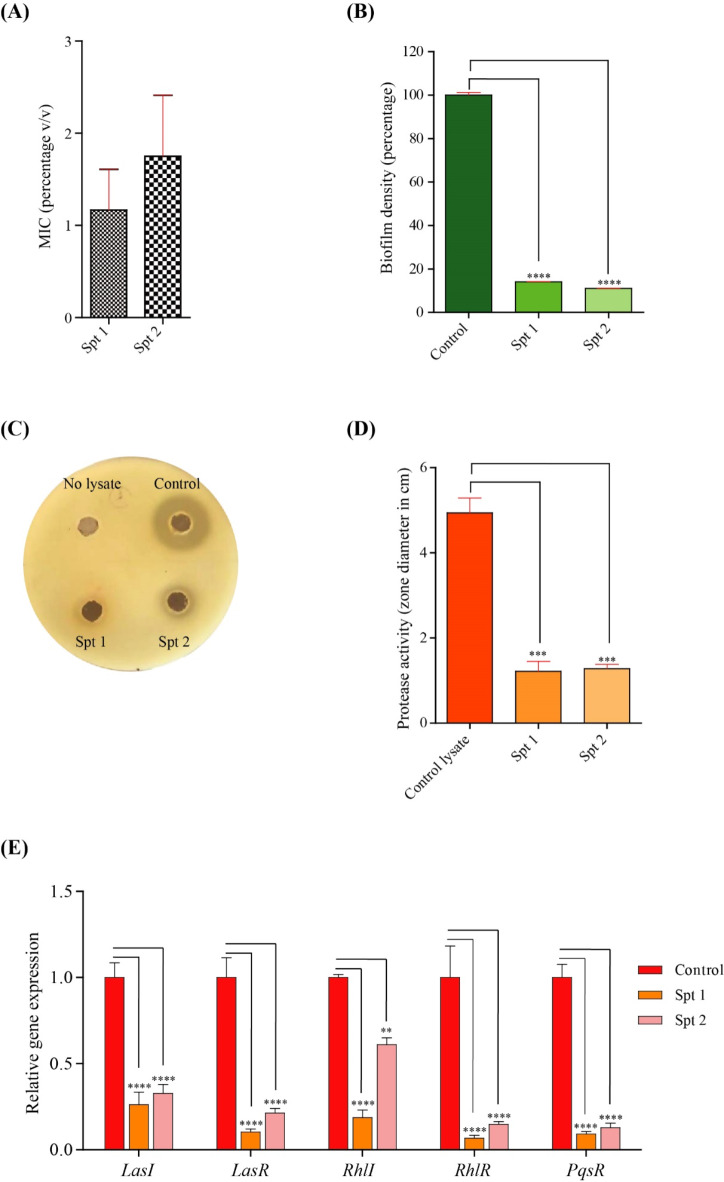
Anti-virulence activity of *S. trilobata* EOs against *P. aeruginosa*. (**A**) MIC (% *v/v*) of Spt 1 and Spt 2, (**B**) Effect of Spt 1 and Spt 2 (1/8th MIC) on biofilm formation, *P. aeruginosa* were cultured in presence of 1/8th MIC of each oil, and a control culture (no oil) was tested in the same experiment. Biofilm density was measured for test and control wells and calculated and graphed as percentage of the Control. (**C**,** D**) Antiprotease activity of Spt 1 and Spt 2 using skim milk agar method, lysate from non-treated culture was used as a control (proficient protease activity), and as a negative control for protease activity we specified one well with no lysate. Diameter of the inhibition zone around the well was measured (in cm) and plotted. (**E**) Evaluating the impact of *S. trilobata* EOs on expression of virulence genes by qPCR. The data represent the mRNA expression of each gene (*LasI*, *LasR*, *RhlI*, *RhlR*, and *PqsR*) relative to *RopD* (housekeeping) and then normalized to the level of the Control. Data were graphed as mean ± SEM from three independent experiments. Two-tailed unpaired Student’s t-test was employed to analyze significance; ** *p* < 0.01, *** *p* < 0.001, **** *p* < 0.0001.

The antimicrobial and anti-virulence activities of the EOs of *S. trilobata* against *P. aeruginosa* were investigated. Initially, the MIC for each oil using the broth microdilution method was determined. Both oils inhibited the growth of *P. aeruginosa* at comparable concentrations, with MIC values of 1.167 ± 0.44% *v/v* for Spt 1 and 1.750 ± 0.6614% *v/v* for Spt 2 (Fig. 3A).

Next, the antibiofilm activities of both EOs were evaluated at sub-MIC concentrations (1/8th of the MIC). Treatment with low doses of the EOs significantly impaired *P. aeruginosa*’s ability to form intact and dense biofilms, resulting in an 83–85% reduction in biofilm formation for both Spt 1 and Spt 2. This demonstrated the remarkable efficacy of the EOs in disrupting microbial biofilm formation (Fig. 3B).

Furthermore, their effect on *Pseudomonas* protease activity was inspected. The ability of lysates from control cultures or oil-exposed cultures to lyse milk casein was tested. While control lysates preserved efficient protease activity, as evidenced by a clear, prominent zone around the well (Fig. 3C). In contrast, cultures treated with the EOs showed a significant loss of protease activity, with a 5-fold reduction in the inhibition zone (Fig. 3D).

Finally, an in-depth investigation into the effect of *S. trilobata* EOs on quorum-sensing genes using qPCR was conducted. The analysis involved measuring the expression profiles of five essential genes (*LasI*, *LasR*, *RhlI*, *RhlR*, and *PqsR*) involved in the quorum sensing machinery. It was revealed that there was a significant reduction in the expression of the inspected genes compared to controls cultured (Fig. 3E). It is worth mentioning that in most cases, the flower heads’ EO (Spt 1) exhibited a more prominent effect on quorum sensing genes compared to the EO of the collected leaves and stems mixture (Spt 2).

### Molecular Docking

Finally, we performed in silico analysis to investigate the virtual binding and affinity of the 4 prominent active components of *S. trilobata* EOs ((+)-(R)-limonene, (±)-α-pinene, α-phellandrene, and α-thujene) against five crucial proteins involved in biofilm, quorum sensing, and protease activity of *P. aeruginosa*. Molecular docking was conducted to reveal each ligand’s binding mode and binding affinity to its corresponding protein active site^21^. Undoubtedly, studying the structure-activity relationship (SAR) of each ligand is a crucial technique for understanding their biological interactions^22^. Given that the tested compounds are cyclic hydrocarbons, their activity is predicted to rely primarily on hydrophobic and hydrophilic interactions rather than H-bonds, which typically require the presence of electronegative atoms. Supplementary Table S2 indicates the collective docking scores for each compound against the 5 selected targets.

**Fig. 4:**
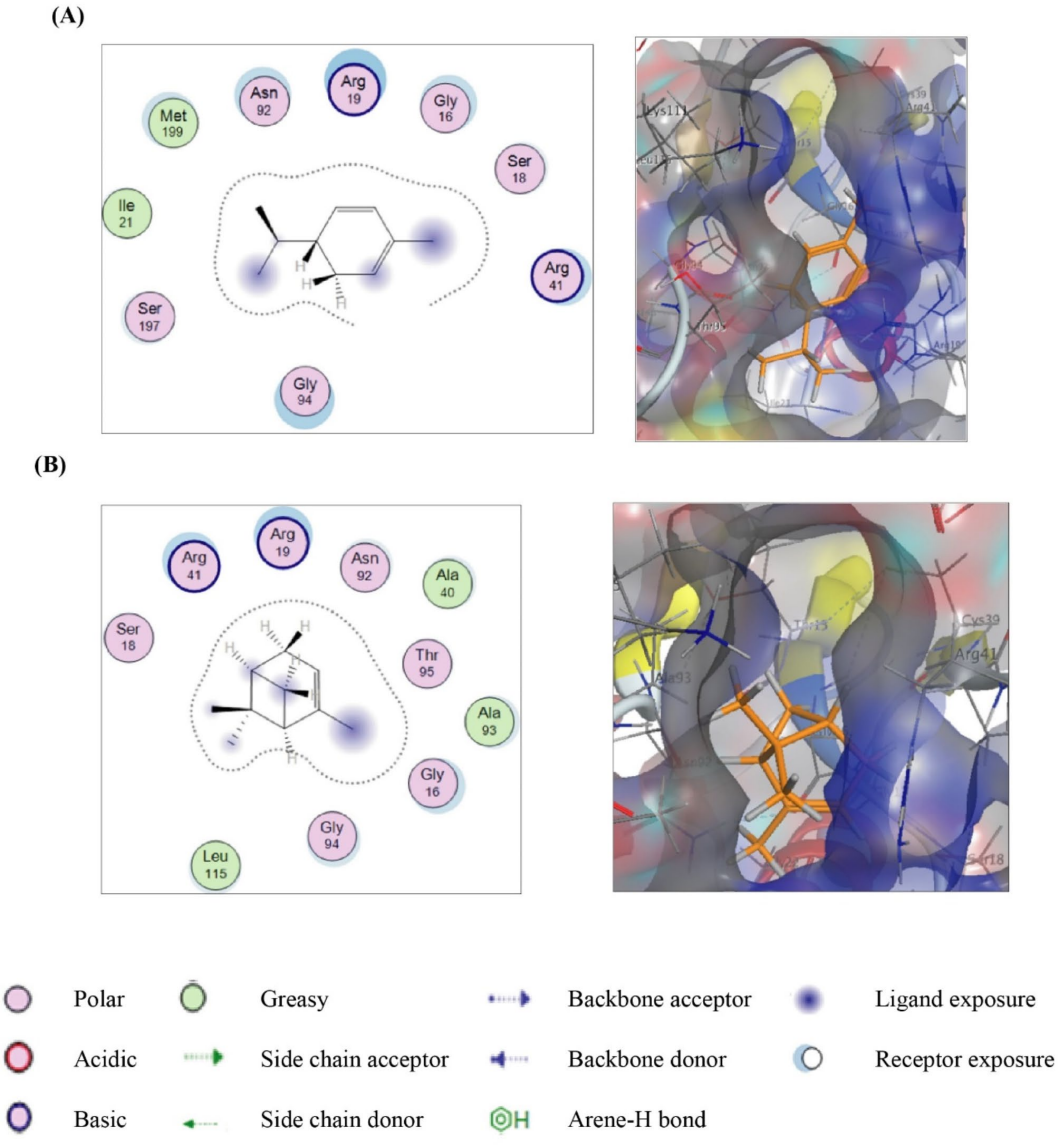
2D and 3D putative interactions of α-phellandrene (upper panel) (**A**) and compounds (±)-α-pinene (lower panel) (**B**) with *P. aeruginosa* RhlG/NADP active-site (PDB: 2B4Q) drawn by Docking suite MOE (Molecular Operating Environment) version MOE 2019.0102,2.

In Fig. 4A, the docking results of α-phellandrene, 5-isopropyl-2-methyl-1,3-cyclohexadiene, against the Nicotinamide-Adenine-Dinucleotide Phosphate (NADP) binding site of the crystal structure of *RhlG* displayed strong hydrophilic/hydrophobic interactions inferred from blue-shaded methyl group at position 2, C-1 in cyclohexadiene moiety and the isopropyl group attached to C-5 from ligand side, and the cyan-shaded conserved amino acids Gly16, Arg19, Arg41, Asn92, Gly94 and Met199 from the receptor side^23,24^. Though, these hydrophobic fitting points inside the pocket of the receptor, which have been created at the initial step of molecular docking, have steered the ligand inside the pocket to form a stable ligand/receptor complex of -7.37377691 Kcal/mol^25^. Such directing hydrophobic points arose from Lennard-Jones potential between a carbon probe and each atom of the residues bordered the binding site^26^. Likewise, docking results of (±)-α-pinene (Fig. 4B), 2,6,6-trimethylbicyclo[3.1.1]hept-2-ene, showed strong hydrophilic/hydrophobic between the three methyl groups, C-4, and C-5 of the ligand and the conserved amino acids Gly16, Arg19, Ala40, Arg41, Ala93, and Gly94, that gave rise to achieve free binding score value of -7.28497887 Kcal/mol.

**Fig. 5:**
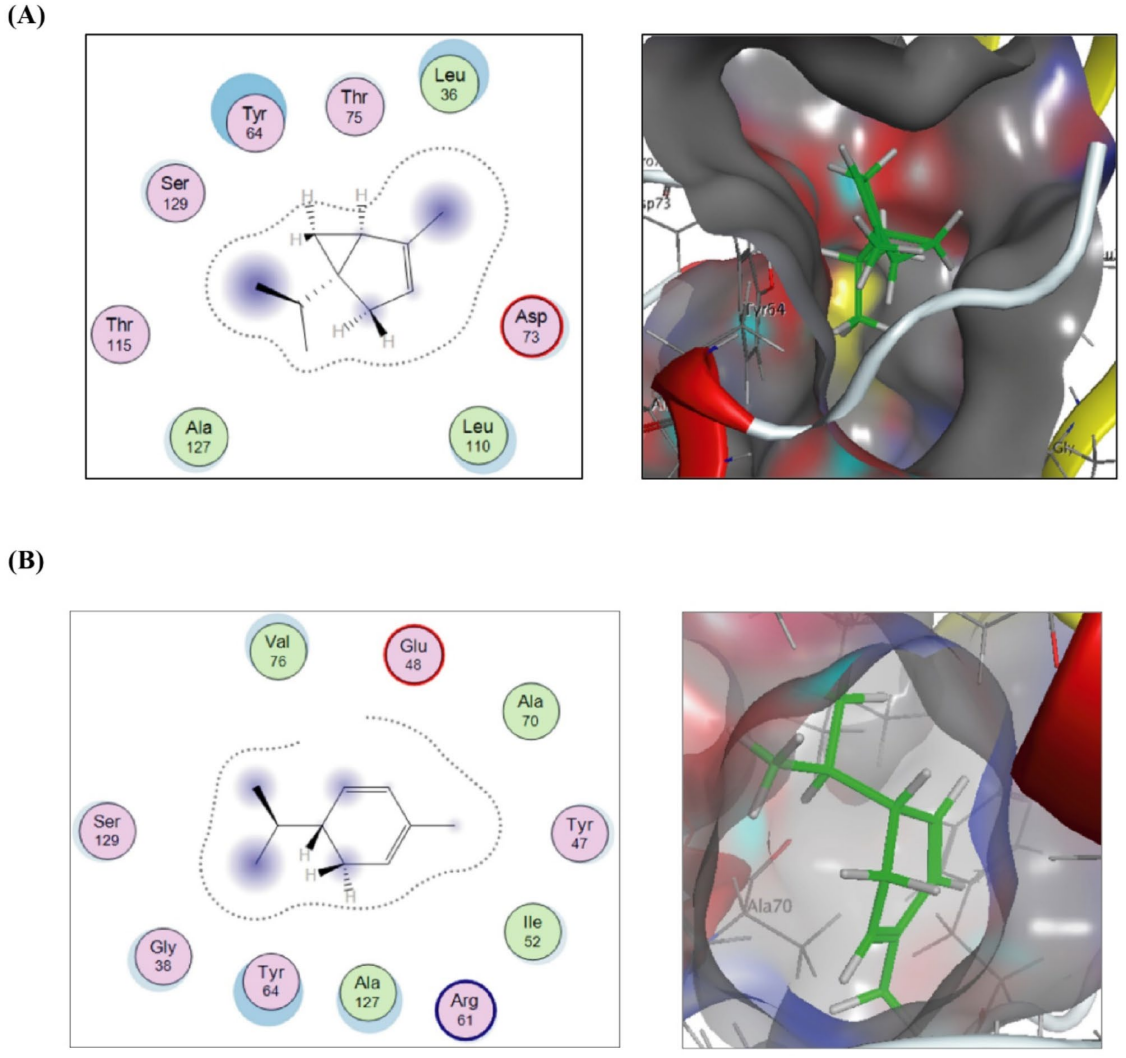
2D and 3D putative interactions of α-thujene (upper panel) (**A**) and compounds α-phellandrene (lower panel) (**B**) with Structure of the *P. aeruginosa LasR* ligand-binding domain bound to its autoinducer (PDB: 2UV0) drawn by Docking suite MOE (Molecular Operating Environment) version MOE 2019.0102,2.

As shown in Fig. 5A, docking of α-thujene, 5-isopropyl-2-methylbicyclo[3.1.0]hex-2-ene, against the crystal structure of the *P. aeruginosa LasR* ligand-binding domain bound to its autoinducer revealed significant hydrophilic and hydrophobic interactions. Specifically, the methyl group at C-2 and the isopropyl group at C-5 interacted with conserved amino acids Leu36, Tyr64, Asp73, Tyr75, Leu110, Ala127, and Ser129, from the pocket side paving the way for the ligand/receptor complex to score free binding energy of -7.63352394 Kcal/mol.

In the lower panel of the same figure (Fig. 5B), α-phellandrene as a hydrocarbon compound displayed through two substituents and C- and C-6 hydrophilic/hydrophobic interactions with Gly38, Tyr47, Tyr64, Val76, Ala127, and Ser129 scoring free energy of binding value of -7.56075001 Kcal/mol.

**Fig. 6:**
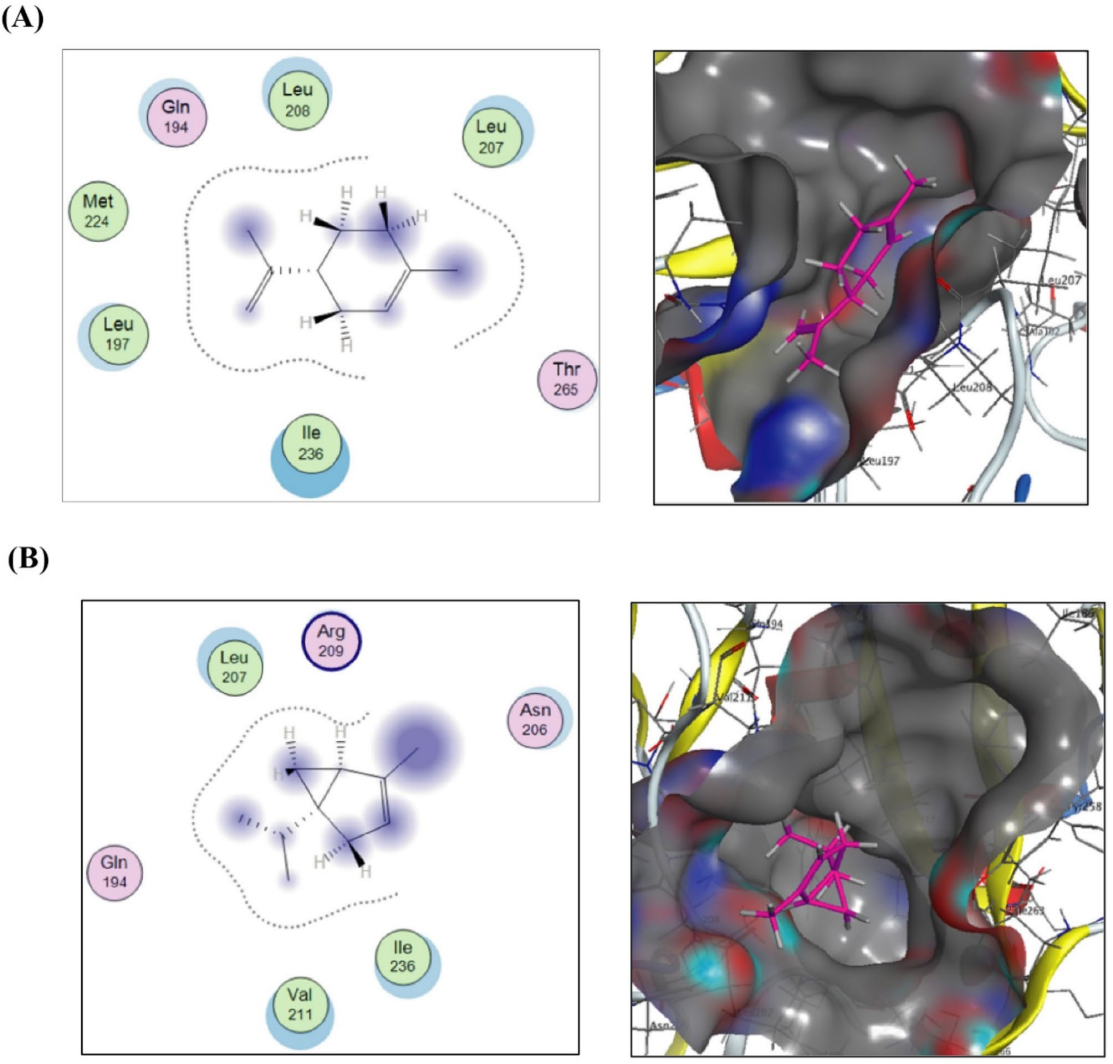
2D and 3D putative interactions of (+)-(R)-limonene (upper panel) (**A**) and compounds α-thujene (lower panel) (**B**) with crystal structure of *PqsR* coinducer binding domain of *P. aeruginosa* with ligand NHQ (PDB: 4JVD) drawn by Docking suite MOE (Molecular Operating Environment) version MOE 2019.0102,2.

In Fig. 6A, docking of (+)-(R)-limonene, (R)-1-methyl-4-(prop-1-en-2-yl)cyclohex-1-ene, onto the crystal structure of *PqsR* coinducer binding domain of *P. aeruginosa* with ligand NHQ, revealed that C-1, C-3, C-4 and the two substituents are involved in the hydrophilic/hydrophobic interactions with the conserved amino acids Gln194, Leu197, Leu207, Leu208, and Ile236 from the receptor side. These interactions resulted in a total free binding energy of -7.38769913 Kcal/mol. In the lower panel of the same figure (Fig. 6B), α-thujene demonstrated hydrophilic and hydrophobic interactions through nearly its entire structure with conserved amino acids Asn206, Leu207, Val211, and Ile236 on the receptor side, achieving a free binding energy value of -6.93545008 Kcal/mol.

**Fig. 7:**
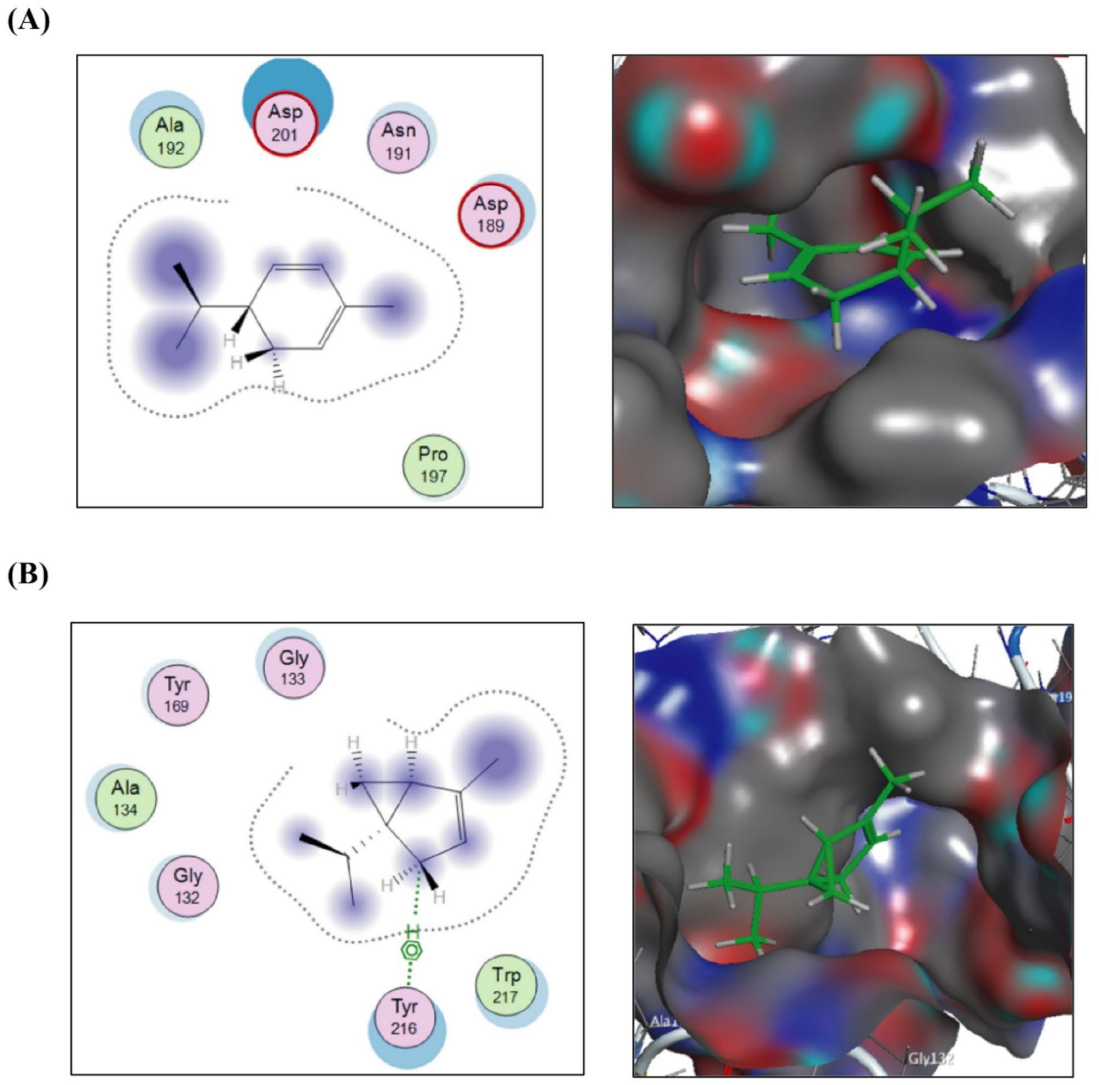
2D and 3D putative interactions of α-phellandrene (upper panel) (**A**) and compounds α-thujene (lower panel) (**B**) with alkaline protease from *P. aeruginosa* IFO3080 (PDB: 1AKL) drawn by Docking suite MOE (Molecular Operating Environment) version MOE 2019.0102,2.

Similarly, as shown in Fig. 7A, docking of α-phellandrene against the crystal structure of alkaline protease from *P. aeruginosa* IFO3080, revealed hydrophilic and hydrophobic interactions involving nearly its entire structure. These interactions occurred with conserved amino acids Asp189, Asn191, Ala192, and Asp201 on the receptor side, resulting in a total free binding energy of -7.21749926 Kcal/mol. Likewise, as shown in the lower panel of the same figure (Fig. 7B), α-thujene exhibited hydrophilic and hydrophobic interactions through its entire structure with conserved amino acids Gly132, Gly133, Ala134, Tyr169, Tyr216, and Trp217 within the binding pocket, achieving a total free binding energy value of -6.93198347 Kcal/mol.

**Fig. 8:**
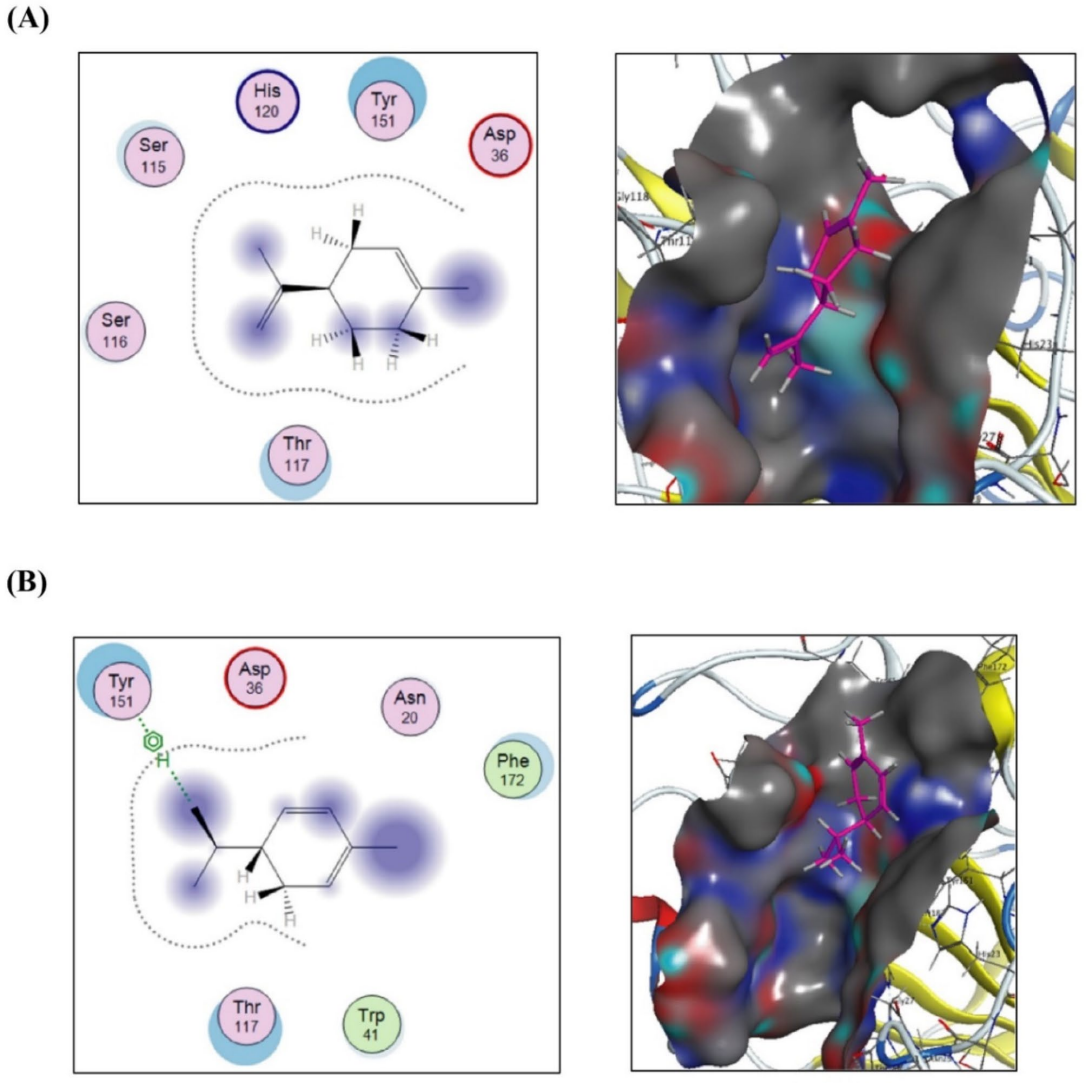
2D and 3D putative interactions of (+)-(R)-limonene (upper panel) (**A**) and compounds α-phellandrene (lower panel) (**B**) with crystal structure of the *LasA* virulence factor from *P. aeruginosa* (PDB: 3IT7) drawn by Docking suite MOE (Molecular Operating Environment) version MOE 2019.0102,2.

Finally, docking of (+)-(R)-limonene onto the crystal structure of the LasA virulence factor from *P. aeruginosa* (PDB: 3IT7) as shown in Fig. 8A, revealed that the two substituents, along with C-3 and C-4, participated in hydrophilic and hydrophobic interactions with conserved amino acids Ser115, Ser116, Thr117, and Tyr151 within the binding pocket. These interactions resulted in a free binding energy value of -6.5292697 Kcal/mol. Additionally, in the lower panel of the same figure (Fig. 8B), C-1, C-3, and C-4 of α-phellandrene, along with its two substituents, engaged in hydrophilic and hydrophobic interactions with conserved amino acids Thr117, Phe172, and Tyr151. Notably, Tyr151, an aromatic amino acid, formed an arene-H bond with the anterior branch of the isopropyl group attached to C-5, further stabilizing the interaction and contributing to a free binding energy of -6.16474915 Kcal/mol.

Notably, as illustrated in the 3D views of the best poses, all tested compounds demonstrated a strong ability to anchor within the binding cavities of the five studied receptors. Among the four tested compounds, the most potent inhibitory activity was observed against the *P. aeruginosa* LasR ligand-binding domain bound to its autoinducer (PDB: 2UV0) and RhlG/NADP active-site complex (PDB: 2B4Q). Specifically, α-phellandrene emerged as the most effective inhibitor of RhlG/NADP active-site complex (PDB: 2B4Q) and alkaline protease (PDB: 1AKL) with free binding energy values of -7.37377691 and − 7.21749926 Kcal/mol respectively. In contrast, α-thujene was identified as the best inhibitor of the LasR ligand-binding domain bound to its autoinducer (PDB: 2UV0) with a free energy of binding value of -7.63352394 Kcal/mol. Meanwhile, (+)-(R)-limonene exhibited the strongest inhibitory activity against PqsR coinducer binding domain of *P. aeruginosa* with ligand NHQ (PDB: 4JVD) and LasA virulence factor (PDB: 3IT7) with with free binding energy values of -7.38769913 and − 6.5292697 Kcal/mol, respectively.

## Discussion

A novel approach to combat microbial resistance is the development of Resistance-Modifying Agents (RMAs), which include antibiotic adjuvants, resistance inhibitors, and antibiotic potentiators. These non-antibiotic compounds are designed to either suppress resistance mechanisms or enhance the effectiveness of existing antibiotics^27–29^. Medicinal plants, phytochemicals, and EOs represent a rich resource for discovering such agents. Many phytochemicals exhibit dual functionality: they possess direct antibacterial effects by disrupting microbial membranes and causing cell leakage, while also acting as resistance modifiers by silencing resistance mechanisms and modulating enzyme activity^10,23,30–33^.

In this study, GC-MS analysis identified 62 components (99.4% of the total) in the EO derived from the leaves and stems mixture (Spt 2) and 43 components (99.64% of the total) in the EO from the flower heads (Spt 1) of *S. trilobata*. Among these, 37 components were common to both EOs. Consistent with previous reports^16,34^, monoterpene hydrocarbons were the predominant chemical class. The major components of Spt 2 were α-thujene (23.94%) and α-pinene (20.75%), while Spt 1 was primarily composed of α-phellandrene (28.30%) and α-pinene (27.25%). Both EOs shared significant amounts of D-limonene (17.66% in Spt 2 and 15.28% in Spt 1). Additionally, Spt 2 contained 25 unique components, including tricyclene, terpinolene, allo-ocimene, cosmen-2-ol, linalool, α-campholenal, pinocarvone, terpinen-4-ol, carvacrol methyl ether, geranial, bornyl acetate, α-limonene diepoxide, carvacrol, 8-hydroxylinalool, trans-α-bergamotene, elemol, germacrene D-4-ol, copaborneol, epiglobulol, alloaromadendrene oxide-(1), 6-isopropenyl-4,8a-dimethyl-1,2,3,5,6,7,8,8a-octahydro-naphthalen-2-ol, kauran-16-ol, hexenyl tiglate (3*Z*-), nonanal and 2-tridecanone. On the other hand, Spt 1 featured distinct components such as α-phellandrene, o-cymene, isothymol methyl ether, and hexahydrofarnesyl acetone, which were not present in Spt 2. The previous report about the composition of Egyptian Spt 1 stated that β-phellandrene (25.65%) is a major component, followed by others as limonene, γ-terpinene, trans-*β*-caryophyllene and *α*-pinene in percentages of 8.93%, 5.90%, 4.83% and 4.72%, respectively^16^. However, no prior studies have documented the EO composition of the leaves and stems mixture. Notably, 27 components were identified for the first time in Spt 1, as detailed in Supplementary Table S1. Additionally, eleven components reported by Koheil^16^ were not found in Spt 1. However, other countries have investigated the EO components of *S. trilobata*. The major oil components of the plant from China were α-phellandrene, limonene and germacrene D^14^. In Brazilian *S. trilobata*, the major EO components were *α*-pinene, *α*-phellandrene, *β*-pinene, limonene and γ-muurolene^35^. Comparing to other species, Dai^34^ stated that limonene and α-pinene were the main volatile components of *Wedelia prostrata*. *W. paludosa* EO was reported to be rich in limonene, γ-muurolene and *β*-pinene^35^. Furthermore, a previous study revealed the predominance of α-pinene, limonene, carvacrol, caryophyllene, spathulenol and sabinene in *W. urticifolia* oil^36^. These differences in EO composition are attributed to factors such as geographical origin, species variation, collection time, extraction methods, and plant organ^12,37^.

*P. aeruginosa*, an opportunistic pathogen, known for causing severe acute and chronic infections, particularly in immunocompromised individuals. Its remarkable persistence in clinical environments stems from its capability to form antibiotic-resistant biofilms^38^. These biofilms consist primarily of autogenic extracellular polymeric substances, which serve as a framework to bind bacteria together on surfaces. They also provide protection against environmental stresses, hinder phagocytosis, and enable colonization and long-term survival^38,39^. A key factor enabling *P. aeruginosa* biofilm formation is its efficient cell-to-cell communication system, known as quorum sensing^40^. According to the literatures, several EOs such as mint, clove, cinnamon, chamomile, rose, and mandarin have been documented for their effective antibiofilm and anti-virulence activities against *P. aeruginosa*^41^. Antibiofilm and anti-virulence activities of the EOs from *S. trilobata* and other *Sphagneticola* species have not been studied yet against *P. aeruginosa*.

In this context, we identified that *S. trilobata* EOs from different plant organs at a concentration of 1–2% *v/v* effectively killed *P. aeruginosa* PAO1. While this MIC range is considered moderate to noteworthy^42^, further evaluation of the oil’s anti-biofilm and anti-quorum sensing activities at sub-inhibitory concentrations demonstrated exceptional anti-virulence properties. The oil disrupted biofilm formation at sub-MIC levels and significantly blocked the protease enzyme essential for anchoring host cells *via* its hydrolytic activity. This reduction in protease activity suggests the EO’s potential to impede bacterial spreading. Similar to the literature^34,43^, the antimicrobial activities of *S. trilobata* are attributed to terpenoid compounds such as α-phellandrene, α-pinene, and D-limonene found in its EOs. In addition, many studies have shown that terpene combinations inhibit biofilm formation in various bacterial species and yeasts^13,44^ through interference with the initial adhesion stages and quorum sensing.

Real-time monitoring of gene clusters regulating quorum sensing^45,46^, which regulate bacterial motility and biofilm formation^47^, demonstrated clear and significant inhibitory effects of *S. trilobata* EO at a sub-MIC dose on quorum sensing gene circuits at the transcriptional level. In conclusion, *S. trilobata* EO is proposed as a natural virulence-mitigating agent against *P. aeruginosa*.

## Methods

### Plant material and hydrodistillation of volatile components

Collection of Freshly leaves and stems mixture (268 g) and flower heads (139 g) of *Sphagneticola trilobata* L. Pruski were in December 2022 from Cairo Festival City, New Cairo, Egypt. Botanical authentication was performed by Prof. Dr. Abdel-Halim Abdel-Mogaly, Centre for Agricultural Research, Egypt. A voucher sample (ST-Co 15) of *S. trilobata* was retained in the Herbarium of the Pharmacognosy Department, Faculty of Pharmacy, Zagazig University, Egypt. The studied organs were separately hydrodistilled by the Clevenger apparatus for 4 h to give a pale-yellow oil with a strong aromatic odour. The yields of the EOs were 0.14% and 0.18% from the freshly collected leaves and stems mixture (Spt 2) and flower heads (Spt 1), respectively. The hydrodistilled oils of *S. trilobata* were collected, dried, and maintained in a dark closed vial in the freezer until analysis.

### GC/MS analysis

Mass spectra were determined using a Shimadzu GCMS-QP2010 [Kyoto, Japan] outfitted with a split-splitless injector and a Rtx-5MS fused bonded column [30 m x 0.25 mm i.d. x 0.25 μm film thickness] from Restek, USA. The column temperature was initially adjusted at 45 °C lasting to 2 min (isothermal) and then programmed to 300 °C (5 °C/min), holding at 300 °C lasting to 5 min (isothermal). The injector temperature was adjusted at 250 °C. The flow rate of helium as a carrier gas was 1.41 ml/min. A filament emission current, ionization voltage, and ion source were set at 60 mA, 70 eV, 200 °C, respectively. EOs samples were diluted with hexane in a percentage of 1% *v/v* and injected in split mode with a 1:15 split ratio.

### Identification and quantification of volatile components

Volatile components were determined by directly comparing of their mass spectra and retention indices (RIs) with a Mass Spectral Library (NIST)^48,49^. Calculation of the content of each peak depended on peak area% relative to a total peak area. In comparison to a homologous series of C_8_–C_28_ n-alkanes injected under the identical circumstances, RIs were determined^50^.

### Bacterial strain, media, and chemicals

The *P. aeruginosa* PAO1 strain used in this study was obtained from the Department of Microbiology, Faculty of Pharmacy, Zagazig University. Microbiological media, including Mueller Hinton (MH) broth, Tryptone soya broth (TSB) and agar (Oxoid, Hampshire, UK). Ciprofloxacin was purchased from Sigma Chemical Co. (St. Louis, MO, United States). All chemicals utilized were of pharmaceutical grade. For each experiment, *S. trilobata* EOs (Spt 1 and Spt 2) were solubilized in the respective media using 1% Tween-20. Control groups from bacterial culture (without adding the EO) were prepared for each experiment.

### MIC determination

The broth dilution method was employed for MIC measurement of *S. trilobata* oil in 96-well plates as described in the reported data^20,31,51^. Two-fold serial dilutions of *S. trilobata* oil, starting from 20% *v/v* in DMSO, were solubilized in Mueller–Hinton broth using 1% Tween-20. Subsequently, 50 µL of a 0.5 MacFarland bacterial suspension was added to each well, and the plate was then incubated overnight at 37 °C. Three wells with 1 µg/mL Ciprofloxacin were marked as positive control (No bacterial growth). The lowest concentration that inhibits visible growth is the MIC.

### Assessment of biofilm Inhibition

Biofilm density was quantified as previously reported^23,31,32^. Briefly, bacterial overnight cultures in TSB were diluted to an OD600 of 0.4. 10 µL aliquots of the bacterial suspensions were added to 10 mL of fresh medium with *S. trilobata* oil (1/8th of the MIC) solubilized in Tween. The plates were then incubated for 24 h at 37 °C. Following incubation, planktonic cells were aspirated, and the plates were softly washed and air dried. The attached bacterial cells were fixed with methanol for 25 min and then stained with 1% crystal violet for 20 min. After removing the excess dye, the bound stain was dissolved in 33% glacial acetic acid, and the absorbance was measured at 590 nm using a Biotek Spectrofluorometer (Biotek, Winooski, VT, USA). The test was performed in triplicate, and the absorbance of *S. trilobata* oil-treated PAO1 was presented as the mean ± SEM of the percentage change from untreated controls.

### Evaluation of protease activity

The skim milk agar (5%) method was employed according to^31,33^. Overnight cultures of PAO1 in TSB, along with *S. trilobata* oil (1/8th of the MIC) solubilized with Tween were centrifuged at 10,000× g for 20 min. Subsequently, aliquots (100 µL) of the supernatants were dispensed into wells created in the agar plates and incubated overnight at 37 °C. The diameters of the clear zones formed around the wells were subsequently measured. The test was conducted in triplicate, and the results were presented as the mean ± SEM of the clear zone diameter for the test and the untreated controls.

### RNA extraction and quantitative Real-Time PCR (qRT-PCR)

RNA extraction was conducted from PAO1 cultures treated and untreated with *S. trilobata* oil (1/8th of the MIC) solubilized with Tween. Briefly, PAO1 cultures were pelleted at 8000 rpm for 10 min at 4 °C. The resulting pellets were re-suspended in 100 µL of Tris-EDTA buffer supplemented with lysozyme and incubated at 25 °C for 5 min. Following incubation, the bacterial pellets were lysed using RNA lysis buffer, and total RNA was isolated and purified according to the GeneJET RNA Purification Kit protocol (ThermoScientific, Waltham, MA, USA). DNase treatment was applied to eliminate any residual chromosomal DNA. The RNA concentrations were then quantified using a NanoDrop ND-1000 spectrophotometer and stored at − 70 °C for future use.

The primers listed in Supplementary Table S3 were employed to evaluate the relative expression of quorum sensing genes in PAO1 strains *via* q-PCR. The cDNA was synthesized using a cDNA Reverse Transcriptase kit (Applied Biosystem, Beverly, MA, USA) and amplified using the PCR Master Kit Syber Green I (Fermentas) with the Step One instrument (Applied Biosystem, Beverly, MA, USA). The PCR amplification process consisted of an initial step of 10 min at 95 °C, followed by 40 cycles of 20 s at 95 °C, 20 s at 62 °C, and 65 s at 72 °C. The housekeeping gene *RopD* was used as a reference to normalize the expression levels of the tested genes, and the relative gene expression was determined using the comparative threshold cycle (ΔΔCt) method, as described previously^31,52^. The experiment was performed in triplicate.

### Molecular docking and ligand receptor interaction analysis

#### Retrieval of macromolecules and ligand

The crystal structures of *P. aeruginosa* oxidoreductase *RhlG/NADP* active-site complex (Å (PDB: 2B4Q)^53^ at a resolution of 2.30 Å, transcriptase *LasR* ligand-binding domain bound to its autoinducer (PDB: 2UV0)^54^ at a resolution of 1.80 Å, transcription regulator *PqsR* coinducer binding domain of with ligand NHQ (PDB: 4JVD)^55^ at a resolution of 2.95 Å, alkaline protease IFO3080 (PDB: 1AKL)^56^ at a resolution of 2.00 Å, and hydrolase *LasA* virulence factor enzyme (PDB: 3IT7)^57^ at a resolution of 2.14 Å, were restored from protein data bank (https://www.rcsb.org/).

The ligands (+)-(R)-limonene, (±)-α-pinene, α-phellandrene, and α-thujene were drawn into drawn into Marvin Sketch of Marvin suite (http://www.chemaxon.com) and the lowest energy three-dimensional conformer for each, was generated then saved as Mol2 format.

#### Preparation of macromolecules

Removal of all water molecules were carried out from the crystal three-dimensional structure of each protein, protonation 3D for each, with their standard geometry, to integrate hydrogen atoms into the protein structure, tracked by energy minimization as program’s default parameters^23,58^.

#### Molecular modeling simulation study

Docking suite MOE (Molecular Operating Environment) version MOE 2019.0102,2^59^ was adopted in running molecular docking study. Each ligand was docked against the rigid binding pocket of the protein using flexible ligand mode. The placement phase generates poses, from ligand conformations. The free energy of binding of the ligand for a certain pose was evaluated using the force field-based scoring function GBVI/WSA ΔG^60^.

## Electronic supplementary material

Below is the link to the electronic supplementary material.

Supplementary Material 1

## Data Availability

All data generated or analysed during this study are included in this published article and its Supplementary Information files.
