# Supplementary material for: Chemical profile, virtual screening, and virulence-inhibiting properties of Sphagneticola trilobata L. essential oils against Pseudomonas aeruginosa
Source: Sci Rep. 2025 Apr 8;15:11964. doi: 10.1038/s41598-025-94486-0 (PMC11978798; doi:10.1038/s41598-025-94486-0)
Supplement: Supplementary file 1 — Supplementary Material 1 [file 41598_2025_94486_MOESM1_ESM.docx]

**Table S1. Components of *S. trilobata*’s EOs using the GC-MS technique**

| No. | Name | M^+^ (*m/z*) | Bp (*m/z*) | RI_R_^(a)^ | Leaves+ Stems | | Flower heads | | Reported Flower heads |
| --- | --- | --- | --- | --- | --- | --- | --- | --- | --- |
|  |  |  |  |  | **RI_Exp_^(b)^** | **% of total** | **RI_Exp_^(b)^** | **% of total** | **% of total^(c)^** |
| *Monoterpene hydrocarbons* | | | | | | | | | |
| 1 | Tricyclene | 136 | 93 | 926 | 919 | 0.05 | - | - | - |
| 2 | *α*-Thujene | 136 | 93 | 930 | 924 | 23.94 | 925 | 0.17 | 1.34 |
| 3 | *α*-Pinene | 136 | 93 | 939 | 938 | 20.75 | 934 | 27.25 | 4.72 |
| 4 | Camphene | 136 | 93 | 954 | 946 | 1.23 | 945 | 0.51 | 0.93 |
| 5 | *β*-Pinene | 136 | 93 | 979 | 975 | 5.42 | 973 | 3.67**^(f)^** | - |
| 6 | Myrcene | 136 | 41 | 990 | 990 | 3.16 | 989 | 1.97**^(f)^** | - |
| 7 | *α*-Phellandrene | 136 | 93 | 1002 | - | - | 1006 | 28.30 | 1.27 |
| 8 | *δ*-3-Carene | 136 | 93 | 1011 | - | - | - | - | 1.00**^(g)^** |
| 9 | *α*-Terpinene | 136 | 121 | 1017 | 1017 | 0.10 | 1015 | 0.12**^(f)^** | - |
| 10 | *o*-Cymene | 134 | 119 | 1026 | - | - | 1023 | 2.33**^(f)^** | - |
| 11 | *D*-Limonene | 136 | 68 | 1029 | 1033 | 17.66 | 1029 | 15.28 | 8.93 |
| 12 | *β*- Phellandrene | 136 | 93 | 1029 | - | - | - | - | 25.65**^(g)^** |
| 13 | *β*-*trans*-Ocimene | 136 | 93 | 1037 | 1038 | 1.12 | 1036 | 0.76 | 0.95 |
| 14 | *β*-*cis*-Ocimene | 136 | 93 | 1050 | 1048 | 1.66 | 1046 | 0.91 | 1.00 |
| 15 | *γ*-Terpinene | 136 | 93 | 1059 | 1058 | 0.08 | 1057 | 0.08 | 5.90 |
| 16 | Terpinolene | 136 | 93 | 1088 | 1086 | 0.08 | - | - | - |
| 17 | (*E*)-4,8-Dimethylnona-1,3,7-triene | 150 | 69 | 1116**^(d)^** | 1114 | 0.38 | 1114 | 0.10**^(f)^** | - |
| 18 | allo-Ocimene | 136 | 121 | 1132 | 1127 | 0.03 | - | - | - |
| Total monoterpene hydrocarbons | | | | | | **75.66** |  | **81.45** | **51.69** |
| *Oxygenated monoterpenes* | | | | | | | | | |
| 19 | Cosmen-2-ol | 152 | 91 | 1090**^(d)^** | 1147 | 0.05 | - | - | - |
| 20 | Linalool | 154 | 71 | 1096 | 1098 | 0.06 | - | - | - |
| 21 | *α*-Campholenal | 152 | 108 | 1126 | 1124 | 0.04 | - | - | - |
| 22 | Pinocarvone | 150 | 53 | 1164 | 1160 | 0.07 | - | - | - |
| 23 | Terpinen-4-ol | 154 | 71 | 1177 | 1175 | 0.03 | - | - | - |
| 24 | Thymol methyl ether | 164 | 149 | 1235 | 1233 | 0.48 | 1233 | 0.40 | 1.12 |
| 25 | Carvacrol methyl ether | 164 | 149 | 1244 | 1242 | 0.06 | - | - | - |
| 26 | Isothymol methyl ether | 164 | 149 | 1245**^(d)^** | - | - | 1229 | 0.02**^(f)^** | - |
| 27 | Geranial | 152 | 69 | 1267 | 1269 | 0.09 | - | - | - |
| 28 | Bornyl acetate | 196 | 95 | 1288 | 1284 | 0.10 | - | - | - |
| 29 | *α*-Limonene diepoxide | 168 | 43 | 1294**^(d)^** | 1250 | 0.18 | - | - | - |
| 30 | Carvacrol | 150 | 135 | 1299 | 1302 | 0.05 | - | - | - |
| 31 | 8-Hydroxylinalool | 170 | 71 | 1367**^(d)^** | 1395 | 0.04 | - | - | - |
| 32 | *trans*-thujen-2-en-ol | 152 | 91 | 1410**^(e)^** | - | - | - | - | 1.22**^(g)^** |
| Total oxygenated monoterpenes | | | | |  | **1.25** |  | **0.42** | **2.34** |
| *Sesquiterpene hydrocarbons* | | | | | | | | | |
| 33 | *α*- Copaene | 204 | 105 | 1376 | 1375 | 0.06 | 1375 | 0.07**^(f)^** | - |
| 34 | *α*- Bourbonene | 204 | 81 | 1383**^(d)^** | - | - | - | - | 0.72**^(g)^** |
| 35 | *β*-Bourbonene | 204 | 81 | 1388 | 1385 | 0.09 | 1384 | 0.06**^(f)^** | - |
| 36 | *β*-Elemene | 204 | 93 | 1391 | 1391 | 0.48 | 1391 | 0.33**^(f)^** | - |
| 37 | Iso-Caryophyllene | 204 | 41 | 1408 | - | - | - | - | 0.88**^(g)^** |
| 38 | *β*-Caryophyllene | 204 | 93 | 1419 | 1422 | 2.67 | 1421 | 2.53 | 4.83 |
| 39 | *β*-Copaene | 204 | 161 | 1432 | 1430 | 0.08 | 1430 | 0.05**^(f)^** | - |
| 40 | *Trans*-*α*-Bergamotene | 204 | 93 | 1434 | 1435 | 0.03 | - | - | 5.28**^(g)^** |
| 41 | *γ-* Elemene | 204 | 121 | 1436 | 1336 | 0.26 | 1336 | 0.17**^(f)^** | - |
| 42 | Aromadendrene | 204 | 41 | 1441 | - | - | - | - | 2.00**^(g)^** |
| 43 | *α*-Humulene | 204 | 93 | 1454 | 1456 | 2.23 | 1455 | 2.06 | 1.72 |
| 44 | Germacrene D | 204 | 161 | 1485 | 1486 | 5.34 | 1484 | 5.01**^(f)^** | - |
| 45 | *α*-Selinene | 204 | 189 | 1498 | - | - | - | - | 1.17**^(g)^** |
| 46 | Bicyclogermacrene | 204 | 121 | 1500 | 1501 | 3.15 | 1499 | 2.88**^(f)^** | - |
| 47 | *α*-Farnesene | 204 | 93 | 1505 | 1506 | 0.45 | 1506 | 0.39**^(f)^** | - |
| 48 | *δ*-Cadinene | 204 | 161 | 1523 | 1524 | 0.38 | 1524 | 0.41**^(f)^** | - |
| Total sesquiterpene hydrocarbons | | | | | | **15.22** |  | **13.96** | **16.6** |
| *Oxygenated sesquiterpenes* | | | | | | | | | |
| 49 | Cubebol | 222 | 161 | 1515 | 1517 | 0.26 | 1516 | 0.14**^(f)^** | - |
| 50 | Elemol | 204 | 59 | 1549 | 1550 | 0.13 | - | - | - |
| 51 | (*E*)-Nerolidol | 222 | 69 | 1563 | 1563 | 0.54 | 1562 | 0.27 | 1.93 |
| 52 | Germacrene D-4-ol | 222 | 81 | 1575 | 1623 | 0.17 | - | - | - |
| 53 | Spathulenol | 220 | 43 | 1578 | 1582 | 1.52 | 1581 | 0.74 | 3.03 |
| 54 | Caryophyllene oxide | 202 | 43 | 1583 | 1587 | 0.56 | 1586 | 0.23 | 2.12 |
| 55 | Copaborneol | 222 | 95 | 1593**^(d)^** | 1608 | 0.12 | - | - | - |
| 56 | Guaiol | 222 | 161 | 1600 | 1600 | 0.08 | 1599 | 0.18**^(f)^** | - |
| 57 | Humulene epoxide 2 | 220 | 67 | 1608 | 1613 | 0.28 | 1613 | 0.20**^(f)^** | - |
| 58 | Epiglobulol | 222 | 43 | 1618**^(d)^** | 1595 | 0.11 | - | - | - |
| 59 | Junenol | 222 | 109 | 1619 | - | - | 1623 | 0.24**^(f)^** | - |
| 60 | *δ*-Cadinol | 222 | 161 | 1620**^(d)^** | - | - | - | - | 2.12**^(g)^** |
| 61 | *τ*-Cadinol | 222 | 161 | 1640 | 1629 | 0.28 | 1628 | 0.16 | 1.83 |
| 62 | Alloaromadendrene oxide-(1) | 220 | 93 | 1641 | 1674 | 0.21 | - | - | - |
| 63 | *τ*-Muurolol | 222 | 95 | 1642 | 1645 | 0.38 | 1644 | 0.25**^(f)^** | - |
| 64 | *α*-Muurolol | 220 | 161 | 1646 | - | - | 1649 | 0.07**^(f)^** | - |
| 65 | *α*-Cadinol | 222 | 95 | 1654 | 1658 | 0.51 | 1657 | 0.36 | 1.02 |
| 66 | Calamenol | 218 | - | 1657**^(d)^** | - | - | - | - | 1.16**^(g)^** |
| 67 | Germacra-4(15),5,10(14)-triene-1-α-ol | 220 | 159 | 1686 | 1690 | 0.23 | 1690 | 0.13**^(f)^** | - |
| 68 | Shyobunol | 222 | 84 | 1689 | 1696 | 0.38 | 1695 | 0.20**^(f)^** | - |
| 69 | 6-Isopropenyl-4,8a-dimethyl-1,2,3,5,6,7,8,8a-octahydro-naphthalen-2-ol | 220 | 159 | 1690**^(d)^** | 1684 | 0.08 | - | - | - |
| 70 | Farnesol | 222 | 81 | 1721**^(d)^** | - | - | - | - | 0.85**^(g)^** |
| 71 | Hexahydrofarnesyl acetone | 250 | 58 | 1845**^(d)^** | - | - | 1834 | 0.10**^(f)^** | - |
| Total oxygenated sesquiterpenes | | | | | | **5.84** |  | **3.27** | **14.06** |
| *Oxygenated diterpenes* | | | | | | | | | |
| 72 | Phytol | 296 | 71 | 1943 | 2108 | 0.68 | 2108 | 0.24**^(f)^** | - |
| 73 | Kauran-16-ol | 290 | 123 | 2210**^(d)^** | 2249 | 0.03 | - | - | - |
| 74 | Kaur-16-en-18-al, (4.*α*.) | 286 | 91 | 2244**^(d)^** | 2277 | 0.16 | 2277 | 0.14**^(f)^** | - |
| Total oxygenated diterpenes | | | | | | **0.87** |  | **0.38** | **-** |
| *Fatty acid ester* | | | | | | | | | |
| 75 | Hexenyl tiglate (*3Z*-) | 182 | 67 | 1317 | 1323 | 0.12 | - | - | - |
| Total fatty acid esters | | | | | | **0.12** |  | **-** | **-** |
| *Saturated hydrocarbons* | | | | | | | | | |
| 76 | Tetracontane | 563 | 57 | 4000**^(d)^** | 3077 | 0.18 | 2918 | 0.16**^(f)^** | - |
| Total saturated hydrocarbons | | | | | | **0.18** |  | **0.16** | **-** |
| *Oxygenated hydrocarbons* | | | | | | | | | |
| 77 | Nonanal | 142 | 57 | 1100 | 1102 | 0.04 | - | - | - |
| 78 | 2-Tridecanone | 198 | 58 | 1495 | 1493 | 0.22 | - | - | - |
| Total oxygenated hydrocarbons | | | | | | **0.26** |  | **-** | **-** |
| Total identified compounds | | | | | | **99.4** |  | **99.64** | **84.69** |

**^(a)^**: Retention indices reported by Adam ^1^; **^(b)^**: Retention indices experimentally determined in comparison to a homologous series of C_8_–C_28_ n-alkanes injected in Rtx-5MS fused bonded column; **^(C)^**: Percentage of volatile components reported by Koheil ^2^; **^(d)^**: Retention indices reported by NIST Standard Reference Data ^3^; **^(e)^**: Retention index reported by Koheil ^2^; **^(f)^**: Components that are found for the first time in the Egyptian flower heads; **^(g)^**: Reported components that are not found in the studied flower heads.

**Table S2. Collective docking scores**

| Compounds | 2B4Q | 2UV0 | 4JVD | 1AKL | 3IT7 |
| --- | --- | --- | --- | --- | --- |
| (+)-(R)-limonene | -7.21870708 | -7.51645947 | -7.38769913 | -6.92374945 | -6.5292697 |
| (±)-α-Pinene | -7.28497887 | -7.1634922 | -6.46736765 | -6.5632596 | -5.87226725 |
| α-Phellandrene | -7.37377691 | -7.56075001 | -6.81996012 | -7.21749926 | -6.16474915 |
| α-Thujene | -7.1473937 | -7.63352394 | -6.93545008 | -6.93198347 | -5.93982649 |

**Table S3. List of the primers used in this study**

| ****Gene**** | ****Forward primer (5′-3′)**** | ****Reverse primer (5′-3′)**** | ****Ref.**** |
| --- | --- | --- | --- |
| ***RopD*** | **CGAACTGCTTGCCGACTT** | **GCGAGAGCCTCAAGGATAC** | **^4,5^** |
| ***LasI*** | **CGCACATCTGGGAACTCA** | **CGGCACGGATCATCATCT** | **^4,5^** |
| ***LasR*** | **CTGTGGATGCTCAAGGACTAC** | **AACTGGTCTTGCCGATGG** | **^4,5^** |
| ***RhlI*** | **GTAGCGGGTTTGCGGATG** | **CGGCATCAGGTCTTCATCG** | **^4,5^** |
| ***RhlR*** | **GCCAGCGTCTTGTTCGG** | **CGGTCTGCCTGAGCCATC** | **^4,5^** |
| ***PqsR*** | **CTGATCTGCCGGTAATTGG** | **ATCGACGAGGAACTGAAGA** | **^4,5^** |

**References**

1 Adams, R. P. Identification of essential oil components by gas chromatography/mass spectrometry. 5 online ed. *Gruver, TX USA: Texensis Publishing* (2017).

2 Koheil, M. Study of the essential oil of the flower-heads of *Wedelia trilobata* (L.) Hitch. *Az. J. Pharm. Sci.* **26**, 288-293 (2000).

3 *NIST Chemistry WebBook, SRD 69*, <https://webbook.nist.gov/chemistry>.

4 El‐Mowafy, S., Shaaban, M. & Abd El Galil, K. Sodium ascorbate as a quorum sensing inhibitor of *Pseudomonas aeruginosa*. *J. Appl. Microbiol.* **117**, 1388-1399 (2014).

5 Fekry, M. *et al.* GC-MS analysis and microbiological evaluation of caraway essential oil as a virulence attenuating agent against *Pseudomonas aeruginosa*. *Molecules* **27**, 8532 (2022).
